# Determination of Methomyl Residue in Tobacco Samples by Heart-Cutting Two-Dimensional Liquid Chromatography with Tandem Mass Spectrometry

**DOI:** 10.1155/2020/8813142

**Published:** 2020-10-13

**Authors:** Shihao Shen, Min Chen, Tiannan Wang, Ting Fei, Dianhai Yang, Miaoling Cao, Da Wu

**Affiliations:** ^1^College of Environmental Science and Engineering, Tongji University, 1239 Siping Road, Shanghai 200092, China; ^2^Technology R&D Center, Shanghai Tobacco Group Co., Ltd., 717 Changyang Road, Shanghai 200082, China

## Abstract

A novel heart-cutting two-dimensional liquid chromatography coupled with tandem mass spectrometry (2D-LC-MS/MS) was developed for the qualitative and quantitative analysis of methomyl residue in tobacco. Compared to traditional methodologies, fairly high sensitivity and stability were achieved, and the sample procedure was simplified in the two-dimensional liquid chromatography (2D-LC) method. Although methomyl had poor retention performance in most of the reversed-phase liquid chromatography (RPLC) columns, an effective RP/RP strategy was successfully facilitated. An XB-Phenyl column was employed in the first dimension to effectively remove thousands of interference compounds in the matrix. In the second dimension, an ADME column was applied for further separation. After optimization of the separation conditions, a six-way valve was utilized for direct transformation of the target fraction from the 1st column to the 2nd column. A dynamic range of 2.5 ng/mL to 500 ng/mL was achieved with correlation coefficient (*r*^2^) greater than 0.9995. The limit of detection and limit of quantification were determined to be 0.69 and 2.30 ng/mL, respectively. The 2D-LC method shows high sensitivity, good reproducibility, and recovery for methomyl in tobacco samples. Therefore, the new method was quite suitable for routine analysis.

## 1. Introduction

Methomyl (*S*-methyl-1-N-[(methylcarbamoyl)oxy]thioacetimidate) is a systemic broad-spectrum carbamate pesticide, which is widely used in agriculture [1]. However, based on acetylcholinesterase inhibition, methomyl was toxic and hazardous to the environment and human beings [2, 3]. Consequently, it was classified as a high hazardous compound by the World Health Organization (WHO) [4]. Therefore, it is important to develop an efficient and sensitive analytical method for trace amount of methomyl residue in complex samples such as tobacco and tobacco products.

Many detection methods of methomyl were reported [4–14], such as liquid chromatography-tandem mass spectrometry (LC-MS/MS) [9–11], HPLC-fluorescence detection [12, 13], and so on. The LC-MS/MS method was widely used for higher sensitivity than the HPLC method. However, there was a serious complex matrix effect during detection of tobacco samples. One effective way was adding extra pretreatment steps to clean up samples. For instance, the QuEChERS (quick, easy, cheap, effective, rugged, and safe) method was used by adding two cleanup steps. However, for tobacco samples, matrix interferences were still serious after QuEChERS in the LC-MS/MS method, which affect ionization efficiency of methomyl. Furthermore, every 15–20 injections would show a visible decrease in intensity of MS. Therefore, a more convenient and efficient method was needed for measurement of methomyl.

Two-dimensional liquid chromatographic (2D-LC) technique provides more separation power and an extended dynamic range for complex samples [15–17]. In our study, a RP/RP-2D-LC-MS/MS method was developed to determine methomyl residue in tobacco, which revealed fairly high selectivity and sensitivity. The new method was achieved by a combination of the XB-Phenyl column and the ADME column. In addition, a tandem spectrometry under a multiple reaction monitoring (MRM) mode was utilized for qualitative and quantitative analysis of methomyl. The approach was engaged for detection of a wide variety of samples.

## 2. Experimental

### 2.1. Apparatus and Chemicals

Two Agilent 1290 Infinity with diode array detector and 2 position/6 port switching valve were from Agilent Technologies Inc. (Agilent Technologies, Santa Clara, USA). AB SCIEX API 4000 Triple-Quad MS with electrospray ion source (AB SCIEX, USA) was utilized in this work.

Ammonium formate and methomyl standard were purchased from Sigma-Aldrich (Sigma-Aldrich, Saint Louis, USA). High performance liquid chromatography (HPLC) grade solvents including methanol (MeOH), acetonitrile (ACN), and formic acid were obtained from Merck (Merck, Darmstadt, Germany). Ultrapure water was obtained by using a Millipore Milli-Q water purification system (USA).

A QuEChERS kit including salt packets and SPE tubes was purchased from Agilent Technologies Inc. PTFE syringe filters (0.22 *μ*m) were also obtained from Agilent Technologies. Tobacco samples were commercial products.

### 2.2. Preparation of Standard Solution

A 2.00 g blank tobacco sample (without methomyl) was weighted into a 50 mL centrifuge tube. The sample was shaken for 1 min after adding 10 mL water and 10 mL ACN. Then, the tobacco sample was pretreated by the simplified QuEChERS method: QuEChERS extraction salts were added, and then the tube was shaken for 2 min and centrifuged at 5000 rpm for 10 min. The obtained solution was filtered with a 0.45 *μ*m PTFE syringe filter.

The 400 *μ*L blank extraction was added to chromatographic bottles and dried up under nitrogen at room temperature. Then, calibration standards solutions were prepared in the chromatographic bottles. Concentrations of the standard solutions were 2.5, 5, 12.5, 62.5, 125, 250, and 500 ng/mL, respectively.

### 2.3. Sample Preparation

**Figure 1:**
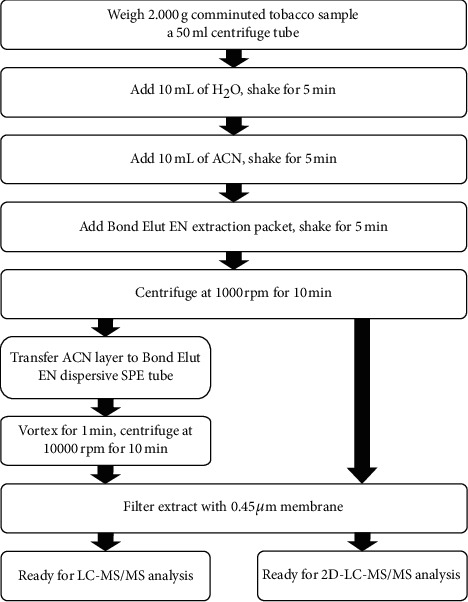
Sample pretreatment processes for LC-MS/MS and 2D-LC-MS/MS analysis.

Methomyl was extracted from the tobacco sample and then cleaned up by a QuEChERS kit (Figure 1). 2.00 g of tobacco powder was weighted in a 50 mL centrifuge tube, 10 mL water was added, and the mixture was shaken for 1 min. Then, 10 mL ACN was added and the mixture was shaken for 2 min. After that, the extraction salt packet was added. The tube was shaken for 2 min and centrifuged at 5000 rpm for 10 min. The ACN layer was filtered and ready for 2D-LC-MS/MS analysis. For LC-MS/MS analysis, 1 mL of the ACN layer was added to the SPE tube for further cleanup. The tube was shaken for 2 min and centrifuged at 5000 rpm for 10 min. The upper layer was filtered and ready for LC-MS/MS analysis.

### 2.4. RP/RP-2D-LC-MS/MS Conditions

First-dimensional separation was as follows: Solvent A1, 0.1% formic acid with 10 mM ammonium formate in 5% MeOH; Solvent B1, 0.1% formic acid with 10 mM ammonium formate in 95% MeOH; 1D column, Welch Ultimate XB-Phenyl column (2.1 × 10 mm, 5 *μ*m); column temperature, 30°C; injection volume, 3 *μ*L.

Second-dimensional separation was as follows: Solvent A2, 0.1% formic acid with 10 mM ammonium formate in 5% MeOH; Solvent B1, 0.1% formic acid with 10 mM ammonium formate in 95% ACN; 2D column, Shiseido CAPCELL Core ADME column (2.1 × 150 mm, 2.7 *μ*m); column temperature, 30°C.

Separation gradients of 1D and 2D chromatography are displayed in Table 1. Valve rotation procedure was as follows: 0–9.1 min, 1D column to waste; 9.1–11.9 min, 1D column to 2D column; 11.9 min, 1D column to waste.

The MS operation conditions were as follows: ion source, ESI; positive scan; MRM mode; ion spray voltage, 5500 V; ion source temperature, 500; curtain gas, 206.7 kPa; Gas 1, 414 kPa; Gas 2, 482.6 kPa; MS/MS acquisition time, 15–20 min. The MS analysis ions of methomyl are shown in Table 2.

## 3. Results and Discussion

### 3.1. The Heart-Cutting 2D-LC-MS/MS System Setup

During determination of methomyl in tobacco by the conventional LC-MS/MS, matrix interference was the major problem which seriously inhibits the signals of the target compound. To reduce the matrix effect, extra cleanup steps were required in the complex samples (Supporting information S1). However, tedious sample preparation steps not only extended the analysis time but also had bad effect on the repeatability and accuracy of the method.

**Figure 2:**
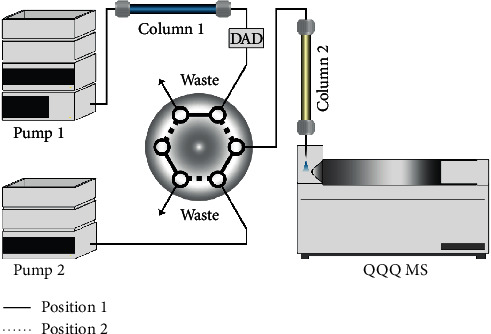
Schematic diagram of the developed 2D-LC-MS/MS system.

In our work, a combination of two-dimensional liquid chromatography was applied to reduce the matrix effect and improve sensitivity of the analyte. The schematic diagram of the developed 2D-LC-MS/MS system is illustrated in Figure 2. The two-dimensional chromatography was coupled with a six-way valve. By the heart-cutting strategy, the interference compounds were effectively removed. Moreover, different stationary phases and mobile phases were employed to increase orthogonality of 2D-LC, and fairly high selectivity was achieved.

### 3.2. Optimization of 2D-LC Separation Conditions

Since retention of methomyl was poor on the C18 column, many other RPLC columns were tried and optimized. Consequently, a XB-Phenyl column and an ADME column were successfully applied in 1D and 2D, respectively. MeOH/water was used as a mobile phase in 1D, while ACN/water was employed in 2D for stronger eluting performance.

**Figure 3:**
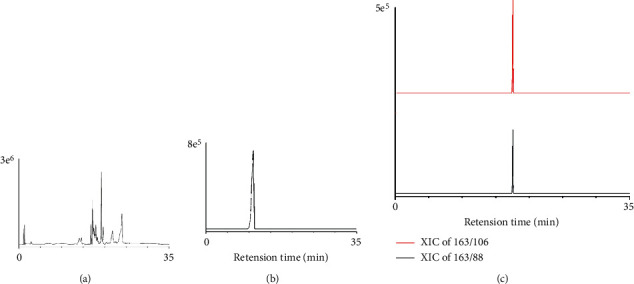
MRM spectrum of the tobacco sample and methomyl standard. (a) 1D-tobacco sample, (b) 1D-methomyl standard, and (c) 2D-tobacco sample.

Heart-cutting was proceeded by the six-way valve shown in Figure 2. There were 3 stages in 2D-LC separation. (1) 1D to the waste: the valve was at position 1. 1D fraction was eluted from the 1D column to the waste. Meanwhile, the 2D column was balanced with the 2D mobile phase. (2) 1D to 2D: the valve was switched to position 2. The methomyl in 1D fraction was flowed to the 2D column. The flow rate of 1D was decreased to 0.2 mL/min, which was a benefit for retention of methomyl on the 2D column. In addition, methomyl was trapped on the 2D column. (3) 2D analysis: the valve was switched back to position 1. At 11.9 min, 2D separation started while other 1D fraction was eluted into waste at the same time. Finally, methomyl was determined qualitatively and quantitatively under MRM mode, and the MRM spectrum is shown in Figure 3.

Methomyl standard was utilized to find out retention time in the 1D separation (Figure 3(b)). The retention time of methomyl was between 9.6 and 11.4 min, so the heart-cutting time range was fixed between 9.1 and 11.9 min. As was shown in Figure 3(a), most of the interference compounds were removed by the heart-cutting strategy. There was almost no interference in determination of methomyl in tobacco samples (Figure 3(c)). Therefore, matrix effect was reduced significantly in MS after 2D separation, and relatively high sensitivity was achieved.

### 3.3. Method Validation

Method validation was studied in detection of methomyl by the 2D-LC-MS/MS method. The developed method showed a wide linear range (from 2.5 to 500 ng/mL) with correlation coefficient (*R*^2^) greater than 0.999. The limit of detection (LOD) and limit of quantification (LOQ) were calculated by signal to noise ratio (S/N). S/N of methomyl for LOD and LOQ was 3 and 10, respectively. LOD and LOQ of the method were 0.69 ng/mL and 2.30 ng/mL, respectively. The recovery of the method was investigated by spiking standard solutions with different concentrations (Table 3). The recovery of methomyl was between 93.1% and 108.2%. RSD of intraday repeatability ranged from 1.9% to 3.3%, and RSD of interday repeatability varied from 2.1% to 5.1%. Therefore, the new method exhibited good linearity, repeatability, and high sensitivity for methomyl analysis.

### 3.4. Comparison with Other Reported Methods

Table 4 summarizes the comparisons of the developed method and other reported methods for the determination of methomyl in different samples. The 2D-LC-MS/MS method revealed fairly high sensitivity and reproducibility. Moreover, the sample pretreatment steps were greatly simplified, which was also a benefit for the analyst and environment [9–21].

## 4. Conclusions

Detection of methomyl residue by conventional LC-MS/MS at extremely low level was difficult because of serious matrix interference in tobacco. A new strategy RPLC/RPLC-MS/MS was employed to effectively reduce the matrix effect. In the separation system, different columns and mobile phases were applied. After first-dimensional separation, most of the interference compounds were removed by heart-cutting. The methomyl fraction was transferred and separated on the second column.

The new method showed fairly high selectivity and sensitivity. LOD and LOQ for methomyl were 0.69 ng/mL and 2.30 ng/mL, respectively. Repeatability of intraday and interday was within 5.1%. In addition, sample procedure steps were simplified because sample purification and enrichment were achieved by the instruments. Furthermore, the system was running fully automatic. Therefore, the new method was quiet suitable for routine analysis. Also, the developed RPLC/RPLC-MS/MS system also provides potential application in analysis of other complex samples.

## Figures and Tables

**Table 1:** 1D-LC and 2D-LC gradient for methomyl analysis.

Time (min)	1D-LC		2D-LC	
	%B	Flow rate (mL/min)	%B	Flow rate (mL/min)
0	5	0.4	5	0.4
3	5	0.4	—	—
8	23	0.4	5	0.4
9	25	0.2	—	—
12	—	—	5	0.2
13	30	0.2	—	—
14	—	—	5	0.4
17	—	—	25	0.4
18	100	0.4	—	—
20	—	—	100	0.4
23	100	0.4	—	—
23.1	5	0.4	—	—
25	—	—	100	0.4
25.1	—	—	5	0.4

**Table 2:** MRM analysis ions of methomyl.

Analyte	Precursor ions (m/*z*)	Product ions (m/*z*)	DP (V)	CE (eV)	Dwell (ms)
Methomyl	163^a^	106	15	9	20
	163^b^	88	44	15	20

^a^Quantitation ion. ^b^Qualitation ion.

**Table 3:** Recovery of methomyl in 2D-LC-MS/MS at different concentrations.

Spiked concentration (ng/mL)	Calculated concentration (ng/mL)	Recovery (%)
50	54.1	108.2
100	98.7	98.7
200	186.2	93.1

**Table 4:** Comparison of the proposed method with other developed methods for the determination of methomyl.

	Method			
Item	LC-MS/MS	LC-MS/MS	LC-MS/MS	2D-LC-MS/MS
Samples	Tea	Citrus fruits	Blood and brain tissue	Tobacco
Sample pretreatment	Matrix solid phase dispersion and concentration	Liquid extraction and concentration	QuEChERS	Simplified QuEChERS
Linear range	1–500 ng/L	0.010–0.150 mg/kg	0.5–500 ng/g	2.5–500 ng/mL
LOD	0.55 ng/g	0.2 *μ*g/kg	0.1 ng/mL in blood and 0.2 ng/g in brain	0.69 ng/mL
RSD	1.3–3.4%	1–11%	4.0–22% for blood and 3.0–14% for brain	5.1%
Recovery	94.8–98.2%	98–103%	90–101% for blood and 84–121% for brain	93.1–108.2%
Ref.	[9]	[11]	[18]	Proposed method

## Data Availability

The data used to support the findings of this study are available from the corresponding author upon request.

## References

[1] Chang C.-F., Chang C.-Y., Hsu K.-E., Lee S.-C., Höll W. (2008). Adsorptive removal of the pesticide methomyl using hypercrosslinked polymers. *Journal of Hazardous Materials*.

[2] El-Demerdash F., Dewer Y., Elmazoudy R. H., Attia A. A. (2013). Kidney antioxidant status, biochemical parameters and histopathological changes induced by methomyl in cd-1 mice. *Experimental and Toxicologic Pathology*.

[3] Xiang G., Li D., Yuan J. (2013). Carbamate insecticide methomyl confers cytotoxicity through dna damage induction. *Food & Chemical Toxicology*.

[4] Numerical List of EHCs No.178 Methomyl, (1996), https://www.who.int/ipcs/publications/ehc/ehc_numerical/en/

[5] Anastassiades M., Lehotay S. J., Štajnbaher D., Schenck F. J. (2003). Fast and easy multiresidue method employing acetonitrile extraction/partitioning and dispersive solid-phase extraction for the determination of pesticide residues in produce. *Journal of AOAC International*.

[6] Yu Y., Liu X., He Z. (2016). Development of a multi-residue method for 58 pesticides in soil using QuEChERS and gas chromatography-tandem mass spectrometry. *Analytical Methods*.

[7] Peng X., Zeng L., Wu C. (2017). Determination of nine mycotoxins and pesticide residues in Xinhui dried orange peel by liquid chromatography-tandem mass spectrometry with Qu ECh ERS clean-up. *Journal of Instrumental Analysis*.

[8] Huang Z., Zhang Y., Wang L. (2009). Simultaneous determination of 103 pesticide residues in tea samples by LC-MS/MS. *Journal of Separation Science*.

[9] Cao Y., Tang H., Chen D., Li L. (2015). A novel method based on MSPD for simultaneous determination of 16 pesticide residues in tea by LC-MS/MS. *Journal of Chromatography B*.

[10] Kruve A., Haapala M., Saarela V. (2011). Feasibility of capillary liquid chromatography-microchip-atmospheric pressure photoionization-mass spectrometry for pesticide analysis in tomato. *Analytica Chimica Acta*.

[11] Fernández R., Garrido Frenich A., Martínez Vidal J. L., Romero González R, Hernández Torres M. E (2009). One-year routine application of a new and rapid method based on ultra performance liquid chromatography-tandem mass spectrometry to the analysis of selected pesticides in citrus fruits. *Analytical Sciences: The International Journal of the Japan Society for Analytical Chemistry*.

[12] Fillion J., Sauvé F., Selwyn J. (2000). Multiresidue method for the determination of residues of 251 pesticides in fruits and vegetables by gas chromatography/mass spectrometry and liquid chromatography with fluorescence detection. *Journal of AOAC International*.

[13] Bache C. A., Lisk D. J. (1965). Determination of organophosphorus insecticide residues using the emission spectrometric detector. *Analytical Chemistry*.

[14] Shamsipur M., Yazdanfar N., Ghambarian M. (2016). Combination of solid-phase extraction with dispersive liquid-liquid microextraction followed by GC-MS for determination of pesticide residues from water, milk, honey and fruit juice. *Food Chemistry*.

[15] García M., Galera M., Uclés S. (2018). Ultrasound-assisted extraction based on QuEChERS of pesticide residues in honeybees and determination by LC-MS/MS and GC-MS/MS. *Analytical and Bioanalytical Chemistry*.

[16] Qi D., Fei T., Liu H., Yao H., Wu D., Liu B. (2017). Development of multiple heart-cutting two-dimensional liquid chromatography coupled to quadrupole-orbitrap high resolution mass spectrometry for simultaneous determination of aflatoxin B1, B2, G1, G2, and ochratoxin A in snus, a smokeless tobacco product. *Journal of Agricultural and Food Chemistry*.

[17] Chen M., Wang L., Dong H. (2017). Quantitative method for analysis of tobacco-specific N -nitrosamines in mainstream cigarette smoke by using heart-cutting two-dimensional liquid chromatography with tandem mass spectrometry. *Journal of Separation Science*.

[18] Horvatovich P., Hoekman B., Govorukhina N., Bischoff R. (2010). Multidimensional chromatography coupled to mass spectrometry in analysing complex proteomics samples. *Journal of Separation Science*.

[19] Shao X., Chen M., Wu D., Liu B., Zhang X., Chen C. (2017). Establishment of a two-dimensional liquid chromatography-tandem mass spectrometry system for detection of four tobacco-specific N-nitrosamines. *Analytical Methods*.

[20] Zhou R., Liu X., Chen L. (2020). Comparison of the antioxidant activities and phenolic content of five Lonicera flowers by HPLC-DAD/MS-DPPH and chemometrics. *International Journal of Analytical Chemistry*.

[21] Dearmond P. D., Brittain M. K., Platoff G. E., Yeung D. T. (2015). Quechers-based approach toward the analysis of two insecticides, methomyl and aldicarb, in blood and brain tissue. *Analytical Methods*.

